# Supplementary material for: Cross-sectional study evaluating data quality of the National Cancer Registration and Analysis Service (NCRAS) prostate cancer registry data using the Cluster randomised trial of PSA testing for Prostate cancer (CAP)
Source: BMJ Open. 2017 Nov 14;7(11):e015994. doi: 10.1136/bmjopen-2017-015994 (PMC5695381; doi:10.1136/bmjopen-2017-015994)
Supplement: Supplementary file 1 [file bmjopen-2017-015994supp001.pdf]

STROBE Statement—Checklist of items that should be included in reports of ***cross-sectional studies***

	Item No	Recommendation
**Title and abstract**	1	*(a)* Indicate the study's design with a commonly used term in the title or the abstract<br>Page 1, Line 6
		(b) Provide in the abstract an informative and balanced summary of what was done and what was found<br>Page 2, Line 3
**Introduction**		
Background/rationale	2	Explain the scientific background and rationale for the investigation being reported<br>Page 5, Line 3-49
Objectives	3	State specific objectives, including any prespecified hypotheses<br>Page 5, Line 53-56
**Methods**		
Study design	4	Present key elements of study design early in the paper<br>Page 6, Line 37
Setting	5	Describe the setting, locations, and relevant dates, including periods of recruitment, exposure, follow-up, and data collection<br>Page 6, Line 6-37
Participants	6	*(a)* Give the eligibility criteria, and the sources and methods of selection of participants<br>Page 6, Line 6-37 & Figure 1
Variables	7	Clearly define all outcomes, exposures, predictors, potential confounders, and effect modifiers. Give diagnostic criteria, if applicable<br>Page 6, Line 59-58 & Page 7, Line 3-37
Data sources/ measurement	8*	For each variable of interest, give sources of data and details of methods of assessment (measurement). Describe comparability of assessment methods if there is more than one group<br>Page 6, Line 6-37
Bias	9	Describe any efforts to address potential sources of bias<br>Page 7, Line 29-37 & Page 8, Line 58 & Page 9, Line 3-4
Study size	10	Explain how the study size was arrived at<br>Page 6, Line 33-37
Quantitative variables	11	Explain how quantitative variables were handled in the analyses. If applicable, describe which groupings were chosen and why<br>Page 6, Line 58 & Page 3, Line 3-10 & Page 7, Line 29-37 Line 54-58 & Page 8, Line 3-4
Statistical methods	12	*(a)* Describe all statistical methods, including those used to control for confounding<br>Page 7, Line 47-58 & Page 8, Line 3-4
		(b) Describe any methods used to examine subgroups and interactions<br>Page 7, Line 54-58 & Page 8, Line 3-4
		(c) Explain how missing data were addressed<br>Page 7, Line 6-10, Line 21-26, Line 30-37
		(*d*) If applicable, describe analytical methods taking account of sampling strategy
		(*e*) Describe any sensitivity analyses
**Results**		
Participants	13*	(a) Report numbers of individuals at each stage of study—eg numbers potentially

		eligible, examined for eligibility, confirmed eligible, included in the study, completing follow-up, and analysed<br>See Figure 1
		(b) Give reasons for non-participation at each stage<br>See Figure 1
		(c) Consider use of a flow diagram<br>See Figure 1
Descriptive data	14*	(a) Give characteristics of study participants (eg demographic, clinical, social) and information on exposures and potential confounders<br>Page 9, Line 5-6
		(b) Indicate number of participants with missing data for each variable of interest<br>Page 9, Line 10-39 & Table 1
Outcome data	15*	Report numbers of outcome events or summary measures<br>Page 9, Line 10-39 & Table 1
Main results	16	*(a)* Give unadjusted estimates and, if applicable, confounder-adjusted estimates and their precision (eg, 95% confidence interval). Make clear which confounders were adjusted for and why they were included<br>Page 9, Line 28-58 & Page 10 Line 3-6 & Tables 2 and 3
		(b) Report category boundaries when continuous variables were categorized<br>Tables 2 and 3
		(*c*) If relevant, consider translating estimates of relative risk into absolute risk for a meaningful time period
Other analyses	17	Report other analyses done—eg analyses of subgroups and interactions, and sensitivity analyses
**Discussion**		
Key results	18	Summarise key results with reference to study objectives<br>Page 14, Line 5-17
Limitations	19	Discuss limitations of the study, taking into account sources of potential bias or imprecision. Discuss both direction and magnitude of any potential bias<br>Page 14, Line 45-53 & Page 15, Line 3-11
Interpretation	20	Give a cautious overall interpretation of results considering objectives, limitations, multiplicity of analyses, results from similar studies, and other relevant evidence<br>Page 15, Line 15-29
Generalisability	21	Discuss the generalisability (external validity) of the study results<br>Page 14 Line 20-42
**Other information**		
Funding	22	Give the source of funding and the role of the funders for the present study and, if applicable, for the original study on which the present article is based<br>Page 16, Line 12-13

*Give information separately for exposed and unexposed groups.

Note: An Explanation and Elaboration article discusses each checklist item and gives methodological background and published examples of transparent reporting. The STROBE checklist is best used in conjunction with this article (freely available on the Web sites of PLoS Medicine at http://www.plosmedicine.org/, Annals of Internal Medicine at http://www.annals.org/, and Epidemiology at http://www.epidem.com/). Information on the STROBE Initiative is available at www.strobe-statement.org.
