# Supplementary material for: Cross-sectional study evaluating data quality of the National Cancer Registration and Analysis Service (NCRAS) prostate cancer registry data using the Cluster randomised trial of PSA testing for Prostate cancer (CAP)
Source: BMJ Open. 2017 Nov 14;7(11):e015994. doi: 10.1136/bmjopen-2017-015994 (PMC5695381; doi:10.1136/bmjopen-2017-015994)
Supplement: Author's manuscript [file bmjopen-2017-015994.draft_revisions.pdf]

Agreement and completeness of prostate cancer data comparing the National Cancer Registration and Analysis Service (NCRAS) and the Cluster randomised triAl of PSA testing for Prostate cancer (CAP)

Journal:	*BMJ Open*
Manuscript ID	bmjopen-2017-015994
Article Type:	Research
Date Submitted by the Author:	19-Jan-2017
Complete List of Authors:	Merriel, Samuel; Univ Bristol, Centre for Academic Primary Care<br>Turner, Emma; University of Bristol, School of Social and Community Medicine<br>Walsh, Eleanor; University of Bristol, School of Social and Community Medicine<br>Young, Grace; University of Bristol, School of Social and Community Medicine<br>Metcalfe, Chris; University of Bristol, Social Medicine<br>Hounsome, Luke; Public Health England South Region, National Cancer Registration and Analysis Service<br>Tudge, Isobel; Public Health England South Region, National Cancer Registry and Analysis Service<br>Donovan, Jenny; University of Bristol<br>Hamdy, Freddie; University of Oxford, Nuffield Department of Surgery<br>Neal, David; University of Oxford Nuffield Department of Surgical Sciences<br>Martin, Richard; University of Bristol, Social Medicine
**Primary Subject Heading**:	Epidemiology
Secondary Subject Heading:	Oncology, Urology, Public health
Keywords:	Prostate disease < UROLOGY, Epidemiology < ONCOLOGY, Urological tumours < ONCOLOGY

Agreement and completeness of prostate cancer data comparing the National Cancer Registration and Analysis Service (NCRAS) and the Cluster randomised triAl of PSA testing for Prostate cancer (CAP)

Dr Samuel WD Merriel[1], Dr Emma L Turner[1], Ms Eleanor Walsh[1], Ms Grace J Young[1], Dr Chris Metcalfe[1], Dr Luke Hounsome[2], Ms Isobel Tudge[2], Prof Jenny Donovan[1], Prof Freddie Hamdy[3], Prof David Neal[3], Prof Richard M Martin[1]

[1]School of Social & Community Medicine, University of Bristol, Bristol, UK

[2]Public Health England National Cancer Registration and Analysis Service, Bristol, UK

[3]Nuffield Department of Surgical Sciences, Oxford University, Oxford, UK

Corresponding author

Dr Samuel William David Merriel

Centre for Academic Primary Care

School of Social & Community Medicine

University of Bristol

39 Whatley Road

Clifton, Bristol, UK

Sam.merriel@bristol.ac.uk

0117 928 9000

Keywords – prostate cancer, registry, validation

Word count – 2059

For peer review only

ABSTRACT (296/300)

Objectives

To compare the completeness and agreement of prostate cancer data recorded by the National Cancer Registration and Analysis Service (NCRAS) with research-level data specifically abstracted from medical records from the Cluster randomised trial of PSA testing for Prostate cancer (CAP) trial.

Design

Cross-sectional comparison study

Participants

We included 1,356 men from the CAP trial cohort who were linked to the NCRAS registry.

Primary and secondary outcome measures

Completeness of prostate cancer data in NCRAS and CAP and agreement for TNM stage (T1/T2; T3; T4/N1/M1) and Gleason grade (4-6; 7; 8-10), measured by differences in proportions and Cohen's Kappa statistic. Data were also stratified by year and pre- versus post-2010, when NCRAS reporting standards changed.

Results

Compared to CAP, completeness was lower in NCRAS for Gleason grade (41.2% vs 76.7%, difference 35.5 95% CI 32.1, 39.0) and TNM stage (29.9% vs 67.6%, difference 37.6 95% CI 34.1, 41.1). NCRAS completeness for Gleason grade (pre- versus post-2010 31.69% vs 64%; difference 32.31 95% CI 26.76, 37.87) and TNM stage (19.31% vs 55.50%; difference 36.19 95% CI 30.72, 41.67) improved over time. Agreement for Gleason grade was high (Cohen's Kappa, κ=0.90 95% CI 0.88, 0.93), but lower for TNM stage (κ=0.41 95% CI 0.37, 0.51) overall. There was a trend towards improved agreement on Gleason grade, but not TNM stage, when comparing pre- and post-2010.

Conclusion

NCRAS data on prostate cancer grade was less complete than CAP. Agreement between the routine registry and research-level data sources for TNM stage was modest. Although the completeness of NCRAS data has improved since 2010, the higher completeness rate in CAP highlights that gains could potentially be achieved

in routine registry data. The modest agreement for TNM stage highlights a need for improved recording of these data in the source medical records.

For peer review only

ARTICLE SUMMARY

Strengths and limitations of this study

- Cross-sectional study design
- Comparison of English prostate cancer registry with independently collected, research-level data from a randomised controlled trial on prostate cancer screening
- Low level of completeness of TNM staging data in NCRAS overall
- Reporting standards for NCRAS registry changed in 2010, affecting the consistency of data collection and quality across the study period

For peer review only

INTRODUCTION

Accurate and complete cancer registry data can inform health policy, prioritise the allocation of limited funds available for cancer prevention and treatment, and be used for research purposes. Bray and Parkin[1,2] outline methods for evaluating the quality of cancer registry data, including comparisons with other local cancer registries, routinely collected data, large-scale research cohorts, or estimates using incidence rates.

Prostate cancer is the most common cancer in males in England, with 39,741 new cases registered in 2014[3]. The National Cancer Registration and Analysis Service (NCRAS) collects data on new cases of cancer in England[4], including prostate cancer. The NCRAS was established in February 2016 to incorporate the National Cancer Registry Service and the National Cancer Intelligence Network, and is now part of Public Health England. The 'Cancer reform strategy'[5] and the 'Improving outcomes: a strategy for cancer'[6] reports from the Department of Health in England highlighted the need for better information and higher quality data to support efforts to improve cancer outcomes. The National Cancer Dataset was subsequently replaced by the Cancer Outcomes and Services Dataset to improve cancer registry data collection standards in England [7]. There is some gray literature on the quality of the NCRAS cancer registry data, showing low levels of completeness of TNM stage[8]. However, to our knowledge, comparison studies against independently collected data have not yet been published for prostate cancer.

The **C**luster randomised tri**A**l of PSA testing for **P**rostate cancer (CAP) trial is a population-based cluster randomized controlled trial (RCT) in England and Wales assessing the impact of a single invitation for PSA testing for prostate cancer on prostate cancer mortality[9]. Within this trial, trained researchers extract detailed data from the medical records of men who have died with or of prostate cancer onto a structured proforma. These data offered the opportunity to compare the completeness and agreement of the NCRAS cancer registry data with an independent research-level data-source.

For peer review only

MATERIALS AND METHODS

Participant selection

NCRAS collate patient-identifiable demographic and cancer specific clinical information based on the Clinical Outcomes and Services Dataset from NHS service providers, unless the patient opts-out.

The CAP trial involves 416,387 men aged 50-69 years from 271 primary care (GP) practices in England and Wales that were randomised between 2001 and 2007[9]. These men were flagged with the Health & Social Care Information Centre (HSCIC) to trace cancer and death registrations. The comparison with the NCRAS data restricts the analysis to those from CAP practices in England. In England, 362,433 CAP men were successfully traced by the HSCIC and 47,556 were notified as having died of any cause. Of the men successfully traced and who had died, 2,111 were identified from HSCIC data as having a prostate cancer diagnosis or having prostate cancer listed anywhere on the death certificate. These men underwent medical record review by specially trained CAP research staff to ascertain cause of death independent from information held on the death certificate and the cancer registry[9]. At the time of this analysis, 1,356 medical record reviews had been completed for men diagnosed with prostate cancer between February 2002 and December 2015, who had died between April 2003 and October 2015. These men were matched to their NCRAS dataset entry using their NHS number (see figure 1), then given a unique identifier and merged.

Figure 1 – Participant recruitment flow diagram

Data

The date of birth provided by the GP practices at the time of recruitment was used to calculate age at entry into the CAP study and age at diagnosis.

The NCRAS team extracted available diagnostic data including date of diagnosis, American Joint Committee on Cancer[10] and Union Internationale Contre le Cancer (UICC) TNM staging[11] and Gleason grade[12] for the current analysis. Clinical TNM

For peer review only

stage was derived from available imaging data, and pathological TNM stage was derived from pathology reports of tissue samples if surgery was within 6 months of diagnosis, with final TNM staging altered if necessary. NCRAS Clinical TNM stage data was combined with pathological stage data where it was incomplete to produce a 'best' stage, and these data were used in the analysis.

The data collected for CAP from the medical records included: date of prostate cancer diagnosis, diagnostic Gleason grade and diagnostic clinical UICC TNM stage. These data were extracted for the current analysis, as well as digital rectal examination (DRE) findings, radiology reports and PSA blood results at the time of diagnosis. The results of these investigations were used in the current analysis to derive clinical TNM stage if this was missing from the data extraction. Diagnostic clinical stage, grade and other investigations were selected by taking the closest record to the date of diagnosis within +/-6 months.

TNM stage data gathered for CAP did not always yield a complete TxNxMx stage from the clinical record. A pragmatic, cascading hierarchical approach was therefore utilised if this situation occurred. Any evidence of advanced disease (T4 or N1 or M1) was gathered initially, followed by evidence of locally advanced disease (T3), then localised disease (T1 or T2). This was done separately for NCRAS and CAP stage data.

Statistical analysis

All analyses were performed using STATA 14 (StataCorp, 2015). We compared the completeness of Gleason grade and TNM stage between NCRAS and CAP data using differences in proportions (with 95% confidence intervals). The agreement between the NCRAS data and CAP data was assessed using Cohen's Kappa statistic, with 95% confidence intervals, for Gleason grading and TNM staging. We also undertook an analysis comparing localised (T1/2) vs locally advanced (T3) vs advanced (T4 or N1 or M1) prostate cancer. Reporting standards for new cases to the registry changed in 2010-2011[7], so the completeness data was also stratified by year of prostate cancer diagnosis and 2002-2009 vs 2010-2015.

For peer review only

RESULTS

The average age at the time of diagnosis of both the NCRAS and CAP participants was 75.2 years (SD 5.1).

97.9% (n=1,327) of men with prostate cancer notified to the NCRAS registry had a complete diagnosis date. Gleason grade was entered for 41.2% (95% CI 38.6, 43.8) of NCRAS men. UICC TNM stage was entered for 29.9% (95% CI 27.5, 32.5). (**See Table 1**).

Of the CAP trial men with complete medical record reviews, 98.5% (n=1,336) had complete date of birth and diagnosis date. The overall completeness of Gleason grade data and TNM stage was 76.7% (95% CI 74.4, 78.9) and 67.6% (95% CI 65.0, 70.0) respectively.

The date of diagnosis in the NCRAS registry exactly matched CAP trial men in 44.5% (n=583) of cases. The median difference in diagnosis date for NCRAS patients was 0 days (interquartile range: 0, 7) later than CAP men. NCRAS had significantly lower overall completeness than CAP for Gleason grade (difference in proportions 35.6% 95% CI 32.1, 39.0), and TNM stage (37.6% 95% CI 34.1, 41.1), although the difference in completeness of Gleason grade (1.00% 95% CI -5.63, 7.63) and TNM stage (11.75% 95% CI 5.05, 18.45) data was much smaller from 2010 onwards.

Table 2 shows agreement on Gleason grade data in the registry and the CAP trial, which was strong. Agreement was high for combined (κ=0.90 95% CI 0.89, 0.92) Gleason grade overall. There was a trend towards higher agreement in 2010-2015 compared to 2002-2009 for low, moderate and high combined Gleason grade. This level of agreement was maintained across individual levels of primary (κ=0.84 95% CI 0.82, 0.86) and secondary Gleason grades (κ=0.80 95% CI 0.79, 0.83).

Table 3 shows agreement on overall UICC TNM staging. The levels of agreement for overall stage accuracy were weak to moderate. Similar levels of agreement were found when considering the data categorized as localised (T1/2 k=0.53 95% CI 0.42,

For peer review only - http://bmjopen.bmj.com/site/about/guidelines.xhtml

0.64), locally advanced (T3 k= 0.29 95% CI 0.17, 0.41) and advanced (T4 or N1 or M1 k=0.47 95% CI 0.37, 0.57). These findings were consistent when comparing data from 2002 – 2009 with 2010 – 2015.

For peer review only

n = 1356	Combined Gleason grade						TNM stage					
	**NCRAS**		**CAP**		**Diff**	**95%CI**	**NCRAS**		**CAP**		**Diff**	**95%CI**
2002-2003	22	26.19%	74	88.10%	-61.91%	-73.58, -50.23	7	8.33%	56	66.67%	-58.33%	-70.02, -46.65
2004	25	33.33%	59	78.67%	-45.34%	-59.47, -31.20	18	24.00%	50	66.67%	-42.67%	-57.06, -28.27
2005	41	33.61%	101	82.79%	-49.18%	-59.91, -38.45	30	24.59%	84	68.89%	-44.26%	-55.48, -33.04
2006	47	29.94%	129	82.17%	-52.23%	-61.57, -42.89	21	13.38%	109	69.43%	-56.05%	-65.01, -47.09
2007	55	31.43%	150	85.71%	-54.28%	-62.90, -45.67	33	18.86%	128	73.14%	-54.29%	-63.04, -45.53
2008	63	34.05%	157	84.86%	-50.81%	-59.37, -42.25	32	17.30%	114	61.62%	-44.32%	-53.20, -35.45
2009	49	31.61%	110	70.97%	-39.36%	-49.58, -29.13	43	27.74%	106	68.39%	-40.65%	-50.81, -30.48
2010	93	62.84%	105	70.95%	-8.11%	-18.79, 2.57	53	35.81%	89	60.16%	-24.32%	-35.36, -13.28
2011	77	68.14%	73	64.60%	3.54%	-8.77, 15.85	51	45.13%	84	74.34%	-29.20%	-41.41, -16.99
2012	66	64.71%	59	57.84%	6.87%	-6.47, 20.20	90	88.24%	69	67.65%	20.59%	9.56, 31.61
2013-2015	20	54.05%	23	62.16%	-8.11%	-30.51, 14.30	28	75.68%	27	72.97%	2.70%	-17.19, 22.60
2002-2009	302	31.69%	780	81.85%	-50.16%	-53.99, -46.32	184	19.31%	647	67.89%	-48.48%	-52.47, -44.70
2010 - 2015	256	64.00%	260	65.00%	-1.00%	-7.63, 5.63	222	55.50%	269	67.25%	-11.75%	-18.45, -5.05
**Overall**	558	41.15%	1040	76.70%	-35.55%	-38.99, -32.09	406	29.94%	916	67.55%	-37.61%	-41.10, -34.12

Table 1 - Completeness of Gleason grade and TNM stage in NCRAS registry and CAP

For peer review only

For peer review only - http://bmjopen.bmj.com/site/about/guidelines.xhtml

n=1356	**NCRAS**	**CAP**	**Kappa**	**95% CI**
Combined Gleason grade (2002 – 2009)				
4-6	80	202	0.84	0.77, 0.92
7	97	241	0.86	0.79, 0.92
8-10	125	337	0.92	0.88, 0.97
Overall	302	780	0.87	0.84, 0.92
Combined Gleason grade (2010 – 2015)				
4-6	37	28	0.95	0.89, 1.00
7	90	93	0.95	0.90, 0.99
8-10	129	139	0.97	0.93, 0.99
Overall	256	260	0.93	0.92, 0.94
Combined Gleason grade (overall)				
4-6	117	230	0.88	0.83, 0.94
7	187	334	0.90	0.86, 0.94
8-10	254	476	0.94	0.92, 0.97
Overall	558	1040	0.90	0.88, 0.93

Table 2 - Agreement between NCRAS registry and CAP on combined Gleason grade

For peer review only

n = 1356	**NCRAS**	**CAP**	**Kappa**	**95% CI**
TNM stage (2002 – 2009)				
T1/T2	91	218	0.55	0.40, 0.69
T3	21	242	0.33	0.15, 0.51
T4/N1/M1	72	122	0.53	0.38, 0.67
Overall	184	582	0.47	0.43, 0.50
TNM stage (2010 – 2015)				
T1/T2	64	57	0.45	0.28, 0.62
T3	40	90	0.25	0.08, 0.41
T4/N1/M1	117	84	0.41	0.28, 0.55
Overall	221	231	0.34	0.27, 0.37
TNM stage (overall)				
T1/T2	155	275	0.53	0.42, 0.64
T3	61	332	0.29	0.17, 0.41
T4/N1/M1	189	206	0.47	0.37, 0.57
Overall	405	813	0.41	0.37, 0.51

Table 3 - Agreement between NCRAS registry and CAP on TNM stage

For peer review only

DISCUSSION

There were mixed levels of completeness and agreement of stage and grade information comparing NCRAS with CAP research-level data. We showed a high level of completeness for diagnosis dates in both cohorts. NCRAS completeness for Gleason grade was moderate and TNM stage completeness was low. From 2010 the completeness of stage and grade data in the NCRAS registry increased. Completeness in CAP remained consistently high throughout the study period. Agreement between NCRAS and CAP was high for Gleason grade and moderate for TNM stage.

Our results are similar to those from validation studies of prostate cancer registries in other countries. Estimates of completeness for registries in Ireland[13], Sweden[14,15], Bulgaria[16], and Norway[17] were generally high, ranging from 91% - 97%. These studies compared registry data with other population registries or primary care records. None of them used an independently collected research-level cancer trial data set for comparison. Tomic et al[15] found a higher level of agreement in Sweden for both TNM staging (83%) and Gleason grading (97%) when compared to our study. This could be due to the lower level of grade and stage completeness in our study and changes in the UICC TNM classification during the study period, and the fact that Tomic et al compared two cancer registries that gathered prostate cancer data on the same population.

The strengths of the data used in the current analysis to compare completeness and agreement with the NCRAS cancer registry were that they were obtained through extensive medical notes review by trained researchers. Further, almost every CAP trial man's diagnosis had been notified to the NCRAS registry, allowing a thorough comparative analysis.

The completeness of UICC TNM stage data in the registry was moderate, making it more difficult to draw conclusions about the accuracy of TNM staging. A possible reason for differences in agreement between NCRAS and CAP could relate to the fact that NCRAS more commonly partially or completely used pathological TNM staging

For peer review only

compared to CAP. Distinguishing TNM stage of prostate cancer, particularly T1b vs T2, is a clinically challenging and controversial area[18], and this may explain some of the reduced reporting. Reporting standards for NCRAS cancer registries changed in 2010 and completeness improved after the changes were made[7].

Our findings suggest the NCRAS prostate cancer registry is complete in terms of identifying and recording prostate cancer diagnoses. This study also demonstrates how trial data can be verified for completeness and accuracy using empirical data. Whilst the agreement for Gleason grade was high, the completeness and agreement of TNM stage data was lower for the years covered in this analysis, highlighting a need for improved recording of these data in the source medical records. Completeness of NCRAS data has improved since 2010, however the higher completeness rate in CAP highlights what further gains could potentially be achieved in routine registry data.

For peer review only

ACKNOWLEDGEMENTS

The authors would like to acknowledge the Public Health England National Cancer Registration and Analysis Service (South West) for their assistance with this study.

FUNDING

The CAP trial was support by Cancer Research UK and the UK Department of Health (C11043/A4286, C18281/A8145, C18281/A11326 and C18281/A15064).

CONFLICTS OF INTEREST

All authors have completed the ICMJE uniform disclosure form at www.icmje.org/coi_disclosure.pdf and declare: no support from any organisation for the submitted work; RM,ET, JOD, and CM have received grants from Cancer Research UK in the previous three years; no other relationships or activities that could appear to have influenced the submitted work.

ETHICAL APPROVAL

Ethical approval to access information about all those in the CAP study was obtained from UK Trent Multi-Centre Research Ethics Committee (MREC/03/4/093). The CAP study was exempt from gaining individual consent to participant having obtained Section 251 approval from the UK Patient Information Advisory Group (PIAG) (now the Confidentiality Advisory Group, CAG), under Section 251 of the NHS Act 2006 [PIAG 4-09 (k)/2003]. The CAP study is sponsored by the University of Bristol and is registered at Current Controlled Trials (ISRCTN92187251).

AUTHORS CONTRIBUTIONS

The CAP trial was performed by R Martin, J Donovan, F Hamdy, R Neal, E Turner, C Metcalfe, G Young and E Walsh. E Turner and E Walsh extracted the relevant CAP trial data for this study. L Hounsome and I Tudge extracted the relevant NCRAS registry data for this study. S Merriel, E Walsh and G Young performed the data analysis. S Merriel drafted this manuscript, to which all authors made amendments and approved the final version.

For peer review only

DATA SHARING STATEMENT

The dataset supporting the conclusions of this article cannot be shared with third parties due to conditions within the Section 251 approval that governs this research.

For peer review only

REFERENCES

1 Bray F, Parkin DM. Evaluation of data quality in the cancer registry: Principles and methods. Part I: Comparability, validity and timeliness. *European Journal of Cancer* 2009;**45**:747–55. doi:10.1016/j.ejca.2008.11.032

2 Parkin DM, Bray F. Evaluation of data quality in the cancer registry: Principles and methods Part II. Completeness. *European Journal of Cancer* 2009;**45**:756–64. doi:10.1016/j.ejca.2008.11.033

3 ONS. Cancer Registration Statistics, England: 2014. 2016;:1–17.

4 NCRAS. National Cancer Research and Analysis Service. httpwww.ncras.nhs.uk.

5 Department of Health. Cancer reform strategy. London: : Department of Health 2007.

6 Department of Health. Improving Outcomes: A Strategy for Cancer. 2011.

7 NCIN. Background to the Cancer Outcomes and Services Dataset (COSD). NCIN 2014.

8 West Midlands Cancer Intelligence Unit. Quantifying the Completeness of National Breast Cancer Data (cases diagnosed in 2006) Executive Summary. Breast Cancer Clinical Outcomes Measures 2009.

9 Turner EL, Metcalfe C, Donovan JL, *et al.* Design and preliminary recruitment results of the Cluster randomised triAl of PSA testing for Prostate cancer (CAP). *British Journal of Cancer* 2014;**110**:2829–36. doi:10.1038/bjc.2014.242

10 AJCC. Prostate Cancer Staging. American Cancer Society 2010.

11 Heidenreich A, Bastian PJ, Bellmunt J, *et al.* EAU Guidelines on Prostate Cancer. Part 1: Screening, Diagnosis, and Local Treatment with Curative Intent—Update 2013. *European Urology* 2014;**65**:124–37. doi:10.1016/j.eururo.2013.09.046

12 Epstein JI. Review Articles An Update of the Gleason Grading System. *JURO* 2010;**183**:433–40. doi:10.1016/j.juro.2009.10.046

13 Kearney TM, Donnelly C, Kelly JM, *et al.* Validation of the completeness and accuracy of the Northern Ireland Cancer Registry. *Cancer Epidemiology* 2015;**39**:401–4. doi:10.1016/j.canep.2015.02.005

14 Barlow L, Westergren K, Holmberg L, *et al.* The completeness of the Swedish Cancer Register – a sample survey for year 1998. *Acta Oncologica* 2009;**48**:27–33. doi:10.1080/02841860802247664

15 Tomic K, Sandin F, Wigertz A, *et al.* Evaluation of data quality in the

For peer review only

National Prostate Cancer Register of Sweden. *European Journal of Cancer* 2015;**51**:101–11. doi:10.1016/j.ejca.2014.10.025

16 Dimitrova N, Parkin DM. Data quality at the Bulgarian National Cancer Registry: An overview of comparability, completeness, validity and timeliness. *Cancer Epidemiology* 2015;**39**:405–13. doi:10.1016/j.canep.2015.03.015

17 Bakken IJ, Gystad SO, Christensen OOS, *et al.* Comparison of data from the Norwegian Patient Register and the Cancer Registry of Norway. *Tidsskrift for Den norske Legeforening* 2012;**132**:1336–40.

18 van Hemelrijck M, Harari D, Garmo H, *et al.* Biomarker-based score to predict mortality in persons aged 50 years and older: a new approach in the Swedish AMORIS study. *International Journal of Molecular Epidemiology and Genetics* 2012;**3**:66–76.

For peer review only

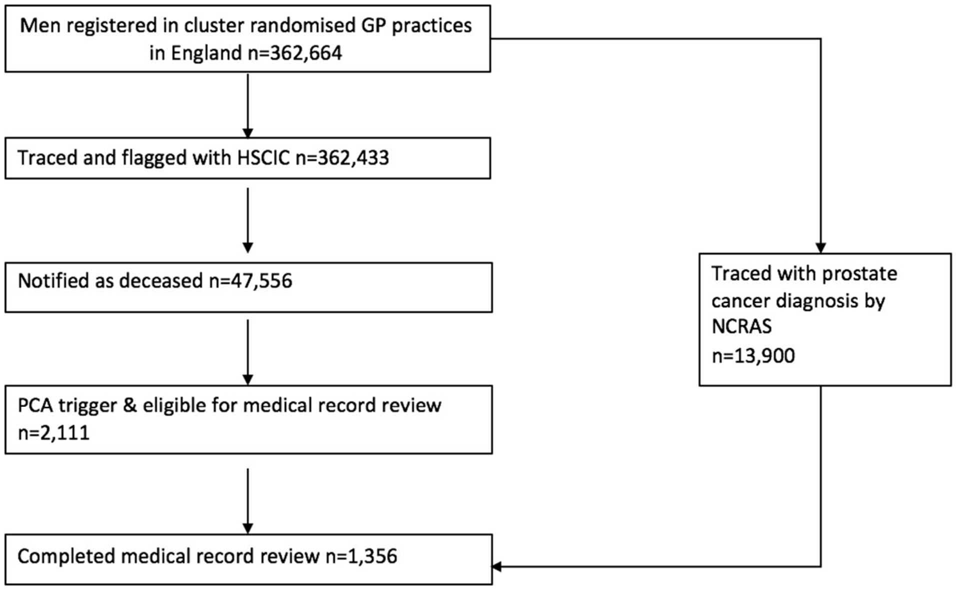

Figure 1 – Participant recruitment flow diagram

192x119mm (144 x 144 DPI)

eview only

Cross-sectional study evaluating data quality of the National Cancer Registration and Analysis Service (NCRAS) prostate cancer registry data using the Cluster randomised triAl of PSA testing for Prostate cancer (CAP)

Journal:	*BMJ Open*
Manuscript ID	bmjopen-2017-015994.R1
Article Type:	Research
Date Submitted by the Author:	20-May-2017
Complete List of Authors:	Merriel, Samuel; University of Bristol School of Social and Community Medicine, Centre for Academic Primary Care<br>Turner, Emma; University of Bristol, School of Social and Community Medicine<br>Walsh, Eleanor; University of Bristol, School of Social and Community Medicine<br>Young, Grace; University of Bristol, School of Social and Community Medicine<br>Metcalfe, Chris; University of Bristol, Social Medicine<br>Hounsome, Luke; Public Health England South Region, National Cancer Registration and Analysis Service<br>Tudge, Isobel; Public Health England South Region, National Cancer Registry and Analysis Service<br>Donovan, Jenny; University of Bristol<br>Hamdy, Freddie; University of Oxford, Nuffield Department of Surgery<br>Neal, David; University of Oxford Nuffield Department of Surgical Sciences<br>Martin, Richard; University of Bristol, Social Medicine
**Primary Subject Heading**:	Epidemiology
Secondary Subject Heading:	Oncology, Urology, Public health
Keywords:	Prostate disease < UROLOGY, Epidemiology < ONCOLOGY, Urological tumours < ONCOLOGY

SCHOLARONE™ Manuscripts

Cross-sectional study evaluating data quality of the National Cancer Registration and Analysis Service (NCRAS) prostate cancer registry data using the Cluster randomised triAl of PSA testing for Prostate cancer (CAP)

Dr Samuel WD Merriel[1], Dr Emma L Turner[1], Ms Eleanor Walsh[1], Ms Grace J Young[1], Dr Chris Metcalfe[1], Dr Luke Hounsome[2], Ms Isobel Tudge[2], Prof Jenny Donovan[1], Prof Freddie Hamdy[3], Prof David Neal[3], Prof Richard M Martin[1]

[1]School of Social & Community Medicine, University of Bristol, Bristol, UK

[2]Public Health England National Cancer Registration and Analysis Service, Bristol, UK

[3]Nuffield Department of Surgical Sciences, Oxford University, Oxford, UK

Corresponding author

Dr Samuel William David Merriel

Centre for Academic Primary Care

School of Social & Community Medicine

University of Bristol

39 Whatley Road

Clifton, Bristol, UK

Sam.merriel@bristol.ac.uk

0117 928 9000

Keywords – prostate cancer, registry, validation

Word count – 2059

For peer review only

ABSTRACT (296/300)

Objectives

To compare the completeness and agreement of prostate cancer data recorded by the National Cancer Registration and Analysis Service (NCRAS) with research-level data specifically abstracted from medical records from the Cluster randomised trial of PSA testing for Prostate cancer (CAP) trial.

Design

Cross-sectional comparison study

Participants

We included 1,356 men from the CAP trial cohort who were linked to the NCRAS registry.

Primary and secondary outcome measures

Completeness of prostate cancer data in NCRAS and CAP and agreement for TNM stage (T1/T2; T3; T4/N1/M1) and Gleason grade (4-6; 7; 8-10), measured by differences in proportions and Cohen's Kappa statistic. Data were also stratified by year and pre- versus post-2010, when NCRAS reporting standards changed.

Results

Compared to CAP, completeness was lower in NCRAS for Gleason grade (41.2% vs 76.7%, difference 35.5 95% CI 32.1, 39.0) and TNM stage (29.9% vs 67.6%, difference 37.6 95% CI 34.1, 41.1). NCRAS completeness for Gleason grade (pre- versus post-2010 31.69% vs 64%; difference 32.31 95% CI 26.76, 37.87) and TNM stage (19.31% vs 55.50%; difference 36.19 95% CI 30.72, 41.67) improved over time. Agreement for Gleason grade was high (Cohen's Kappa, κ=0.90 95% CI 0.88, 0.93), but lower for TNM stage (κ=0.41 95% CI 0.37, 0.51) overall. There was a trend towards improved agreement on Gleason grade, but not TNM stage, when comparing pre- and post-2010.

Conclusion

NCRAS data on prostate cancer grade was less complete than CAP. Agreement between the routine registry and research-level data sources for TNM stage was modest. Although the completeness of NCRAS data has improved since 2010, the higher completeness rate in CAP highlights that gains could potentially be achieved

For peer review only

in routine registry data. The modest agreement for TNM stage highlights a need for improved recording of these data in the source medical records.

For peer review only

ARTICLE SUMMARY

Strengths and limitations of this study

- Cross-sectional study design
- Comparison of English prostate cancer registry with independently collected, research-level data from a randomised controlled trial on prostate cancer screening
- Low level of completeness of TNM staging data in NCRAS overall
- Reporting standards for NCRAS registry changed in 2010, affecting the consistency of data collection and quality across the study period

For peer review only

INTRODUCTION

Accurate and complete cancer registry data can inform health policy, prioritise the allocation of limited funds available for cancer prevention and treatment, and be used for research purposes. Bray and Parkin[1,2] outline methods for evaluating the quality of cancer registry data, including comparisons with other local cancer registries, routinely collected data, large-scale research cohorts, or estimates using incidence rates.

Prostate cancer is the most common cancer in males in England, with 39,741 new cases registered in 2014[3]. The National Cancer Registration and Analysis Service (NCRAS) collects data on new cases of cancer in England[4], including prostate cancer. The NCRAS was established in February 2016 to incorporate the National Cancer Registry Service and the National Cancer Intelligence Network, and is now part of Public Health England. The 'Cancer reform strategy'[5] and the 'Improving outcomes: a strategy for cancer'[6] reports from the Department of Health in England highlighted the need for better information and higher quality data to support efforts to improve cancer outcomes. The National Cancer Dataset was subsequently replaced by the Cancer Outcomes and Services Dataset to improve cancer registry data collection standards in England [7]. There is some gray literature on the quality of the NCRAS cancer registry data, showing low levels of completeness of TNM stage[8]. However, to our knowledge, comparison studies against independently collected data have not yet been published for prostate cancer.

The **C**luster randomised tri**A**l of PSA testing for **P**rostate cancer (CAP) trial is a population-based cluster randomized controlled trial (RCT) in England and Wales assessing the impact of a single invitation for PSA testing for prostate cancer on prostate cancer mortality[9]. Within this trial, trained researchers extract detailed data from the medical records of men who have died with or of prostate cancer onto a structured proforma. These data offered the opportunity to compare the completeness and agreement of the NCRAS cancer registry data with an independent research-level data-source.

MATERIALS AND METHODS

Participant selection

NCRAS collate patient-identifiable demographic and cancer specific clinical information based on the Clinical Outcomes and Services Dataset from NHS service providers, unless the patient opts-out.

The CAP trial involves 416,387 men aged 50-69 years from 271 primary care (GP) practices in England and Wales that were randomised between 2001 and 2007[9]. These men were flagged with the Health & Social Care Information Centre (HSCIC) to trace cancer and death registrations. The comparison with the NCRAS data restricts the analysis to those from CAP practices in England who were deceased. In England, 362,433 CAP men were successfully traced by the HSCIC and 47,556 were notified as having died of any cause. Of the men successfully traced and who had died, 2,111 were identified from HSCIC data as having a prostate cancer diagnosis or having prostate cancer listed anywhere on the death certificate. These men underwent medical record review by specially trained CAP research staff to ascertain cause of death independent from information held on the death certificate and the cancer registry[9]. At the time of this analysis, 1,356 medical record reviews had been completed for men diagnosed with prostate cancer between February 2002 and December 2015, who had died between April 2003 and October 2015. These men were matched to their NCRAS dataset entry using their NHS number (see figure 1), then given a unique identifier and merged.

Figure 1 – Participant recruitment flow diagram

Data

The date of birth provided by the GP practices at the time of recruitment was used to calculate age at entry into the CAP study and age at diagnosis.

The NCRAS team extracted available diagnostic data including date of diagnosis, American Joint Committee on Cancer[10] and Union Internationale Contre le Cancer (UICC) TNM staging[11] and Gleason grade[12] for the current analysis. Clinical TNM

For peer review only

stage was derived from available imaging data, and pathological TNM stage was derived from pathology reports of tissue samples if surgery was within 6 months of diagnosis, with final TNM staging altered if necessary. NCRAS Clinical TNM stage data was combined with pathological stage data by the NCRAS team where it was incomplete to produce a 'best' stage, and these data were used in the analysis.

The data collected for CAP from the medical records included: date of prostate cancer diagnosis, diagnostic Gleason grade and diagnostic clinical UICC TNM stage. These data were extracted for the current analysis, as well as digital rectal examination (DRE) findings, radiology reports and PSA blood results at the time of diagnosis. The results of these investigations were used in the current analysis to derive clinical TNM stage if this was missing from the data extraction. Diagnostic clinical stage, grade and other investigations were selected by taking the closest record to the date of diagnosis within +/-6 months.

TNM stage data gathered for CAP did not always yield a complete TxNxMx stage from the clinical record. A pragmatic, cascading hierarchical approach was therefore utilised if this situation occurred. Any evidence of advanced disease (T4 or N1 or M1) was gathered initially, followed by evidence of locally advanced disease (T3), then localised disease (T1 or T2). This was done separately for NCRAS and CAP stage data.

This study follows the STROBE reporting guidelines for observational studies (STROBE checklist submitted as Supplementary file).

Statistical analysis

All analyses were performed using STATA 14 (StataCorp, 2015). We compared the completeness of Gleason grade and TNM stage between NCRAS and CAP data using differences in proportions (with 95% confidence intervals). The agreement between the NCRAS data and CAP data was assessed using Cohen's Kappa statistic, with 95% confidence intervals, for Gleason grading and TNM staging. We also undertook an analysis comparing localised (T1/2) vs locally advanced (T3) vs advanced (T4 or N1 or M1) prostate cancer. Reporting standards for new cases to the registry changed in

For peer review only

2010-2011[7], so the completeness data was also stratified by year of prostate cancer diagnosis and 2002-2009 vs 2010-2015.

For peer review only

RESULTS

The average age at the time of diagnosis of both the NCRAS and CAP participants was 75.2 years (SD 5.1).

97.9% (n=1,327) of men with prostate cancer notified to the NCRAS registry had a complete diagnosis date. Gleason grade was entered for 41.2% (95% CI 38.6, 43.8) of NCRAS men. UICC TNM stage was entered for 29.9% (95% CI 27.5, 32.5). (**See Table 1**).

Of the CAP trial men with complete medical record reviews, 98.5% (n=1,336) had complete date of birth and diagnosis date. The overall completeness of Gleason grade data and TNM stage was 76.7% (95% CI 74.4, 78.9) and 67.6% (95% CI 65.0, 70.0) respectively.

The date of diagnosis in the NCRAS registry exactly matched CAP trial men in 44.5% (n=583) of cases. The median difference in diagnosis date for NCRAS patients was 0 days (interquartile range: 0, 7) later than CAP men. NCRAS had significantly lower overall completeness than CAP for Gleason grade (difference in proportions 35.6% 95% CI 32.1, 39.0), and TNM stage (37.6% 95% CI 34.1, 41.1), although the difference in completeness of Gleason grade (1.00% 95% CI -5.63, 7.63) and TNM stage (11.75% 95% CI 5.05, 18.45) data was much smaller from 2010 onwards.

Table 2 shows agreement on Gleason grade data in the registry and the CAP trial, which was strong. Agreement was high for combined (κ=0.90 95% CI 0.89, 0.92) Gleason grade overall. There was a trend towards higher agreement in 2010-2015 compared to 2002-2009 for low, moderate and high combined Gleason grade. This level of agreement was maintained across individual levels of primary (κ=0.84 95% CI 0.82, 0.86) and secondary Gleason grades (κ=0.80 95% CI 0.79, 0.83).

Table 3 shows agreement on overall UICC TNM staging. The levels of agreement for overall stage accuracy were weak to moderate. Similar levels of agreement were found when considering the data categorized as localised (T1/2 k=0.53 95% CI 0.42,

For peer review only

0.64), locally advanced (T3 k= 0.29 95% CI 0.17, 0.41) and advanced (T4 or N1 or M1 k=0.47 95% CI 0.37, 0.57). These findings were consistent when comparing data from 2002 – 2009 with 2010 – 2015.

For peer review only

n = 1356	Combined Gleason grade						TNM stage					
	**NCRAS**		**CAP**		**Diff**	**95%CI**	**NCRAS**		**CAP**		**Diff**	**95%CI**
2002-2003	22	26.19%	74	88.10%	-61.91%	-73.58, -50.23	7	8.33%	56	66.67%	-58.33%	-70.02, -46.65
2004	25	33.33%	59	78.67%	-45.34%	-59.47, -31.20	18	24.00%	50	66.67%	-42.67%	-57.06, -28.27
2005	41	33.61%	101	82.79%	-49.18%	-59.91, -38.45	30	24.59%	84	68.89%	-44.26%	-55.48, -33.04
2006	47	29.94%	129	82.17%	-52.23%	-61.57, -42.89	21	13.38%	109	69.43%	-56.05%	-65.01, -47.09
2007	55	31.43%	150	85.71%	-54.28%	-62.90, -45.67	33	18.86%	128	73.14%	-54.29%	-63.04, -45.53
2008	63	34.05%	157	84.86%	-50.81%	-59.37, -42.25	32	17.30%	114	61.62%	-44.32%	-53.20, -35.45
2009	49	31.61%	110	70.97%	-39.36%	-49.58, -29.13	43	27.74%	106	68.39%	-40.65%	-50.81, -30.48
2010	93	62.84%	105	70.95%	-8.11%	-18.79, 2.57	53	35.81%	89	60.16%	-24.32%	-35.36, -13.28
2011	77	68.14%	73	64.60%	3.54%	-8.77, 15.85	51	45.13%	84	74.34%	-29.20%	-41.41, -16.99
2012	66	64.71%	59	57.84%	6.87%	-6.47, 20.20	90	88.24%	69	67.65%	20.59%	9.56, 31.61
2013-2015	20	54.05%	23	62.16%	-8.11%	-30.51, 14.30	28	75.68%	27	72.97%	2.70%	-17.19, 22.60
2002-2009	302	31.69%	780	81.85%	-50.16%	-53.99, -46.32	184	19.31%	647	67.89%	-48.48%	-52.47, -44.70
2010 - 2015	256	64.00%	260	65.00%	-1.00%	-7.63, 5.63	222	55.50%	269	67.25%	-11.75%	-18.45, -5.05
**Overall**	558	41.15%	1040	76.70%	-35.55%	-38.99, -32.09	406	29.94%	916	67.55%	-37.61%	-41.10, -34.12

Table 1 - Completeness of Gleason grade and TNM stage in NCRAS registry and CAP (n, %)

For peer review only - http://bmjopen.bmj.com/site/about/guidelines.xhtml

n=1356	**NCRAS (n)**	**CAP (n)**	**Kappa (k)**	**95% CI**
Combined Gleason grade (2002 – 2009)				
4-6	80	202	0.84	0.77, 0.92
7	97	241	0.86	0.79, 0.92
8-10	125	337	0.92	0.88, 0.97
Overall	302	780	0.87	0.84, 0.92
Combined Gleason grade (2010 – 2015)				
4-6	37	28	0.95	0.89, 1.00
7	90	93	0.95	0.90, 0.99
8-10	129	139	0.97	0.93, 0.99
Overall	256	260	0.93	0.92, 0.94
Combined Gleason grade (overall)				
4-6	117	230	0.88	0.83, 0.94
7	187	334	0.90	0.86, 0.94
8-10	254	476	0.94	0.92, 0.97
Overall	558	1040	0.90	0.88, 0.93

Table 2 - Agreement between NCRAS registry and CAP on combined Gleason grade

For peer review only

n = 1356	**NCRAS (n)**	**CAP (n)**	**Kappa (k)**	**95% CI**
TNM stage (2002 – 2009)				
T1/T2	91	218	0.55	0.40, 0.69
T3	21	242	0.33	0.15, 0.51
T4/N1/M1	72	122	0.53	0.38, 0.67
Overall	184	582	0.47	0.43, 0.50
TNM stage (2010 – 2015)				
T1/T2	64	57	0.45	0.28, 0.62
T3	40	90	0.25	0.08, 0.41
T4/N1/M1	117	84	0.41	0.28, 0.55
Overall	221	231	0.34	0.27, 0.37
TNM stage (overall)				
T1/T2	155	275	0.53	0.42, 0.64
T3	61	332	0.29	0.17, 0.41
T4/N1/M1	189	206	0.47	0.37, 0.57
Overall	405	813	0.41	0.37, 0.51

Table 3 - Agreement between NCRAS registry and CAP on TNM stage

For peer review only

DISCUSSION

There were mixed levels of completeness and agreement of stage and grade information comparing NCRAS with CAP research-level data. We showed a high level of completeness for diagnosis dates in both cohorts. NCRAS completeness for Gleason grade was moderate and TNM stage completeness was low. From 2010 the completeness of stage and grade data in the NCRAS registry increased. Completeness in CAP remained consistently high throughout the study period. Agreement between NCRAS and CAP was high for Gleason grade and moderate for TNM stage.

The completeness of case capture and diagnosis date are consistent with the UK and Ireland Association of Cancer Registries (UKIACR) most recent performance indicators.[13] Our results are similar to those from validation studies of cancer registries in other countries. Estimates of completeness for registries in Ireland[14], Sweden[15,16], Bulgaria[17], and Norway[18] were generally high, ranging from 91% - 97%. These studies compared registry data with other population registries or primary care records. None of them used an independently collected research-level cancer trial data set for comparison. Tomic et al[16] found a higher level of agreement in Sweden for both TNM staging (83%) and Gleason grading (97%) when compared to our study. This could be due to the lower level of grade and stage completeness in our study and changes in the UICC TNM classification during the study period, and the fact that Tomic et al compared two cancer registries that gathered prostate cancer data on the same population.

The strengths of the data used in the current analysis to compare completeness and agreement with the NCRAS cancer registry were that they were obtained through extensive medical notes review by trained researchers. Further, almost every CAP trial man's diagnosis had been notified to the NCRAS registry, allowing a thorough comparative analysis.

The completeness of UICC TNM stage data in the registry was moderate, making it more difficult to draw conclusions about the accuracy of TNM staging. A possible

For peer review only

reason for differences in agreement between NCRAS and CAP could relate to the fact that NCRAS more commonly partially or completely used pathological TNM staging compared to CAP. Distinguishing TNM stage of prostate cancer radiologically, particularly T1b vs T2, is a an evolving area[19], and this may explain some of the difficulties in reporting. Reporting standards for NCRAS cancer registries changed in 2010 and completeness improved after the changes were made[7].

Our findings suggest that prostate cancer data in the NCRAS cancer registry is complete in terms of identifying and recording new cases. Whilst the agreement for Gleason grade was high, the completeness and agreement of TNM stage data was lower for the years covered in this analysis, highlighting a need for improved recording of these data in the source medical records. This study also demonstrates how trial data can be verified for completeness and accuracy using empirical data. Completeness of NCRAS data has improved since 2010, however the higher completeness rate in CAP highlights what further gains could potentially be achieved in routine registry data. Complete and accurate national cancer registries are vital in order to inform health policy, healthcare spending, and research.

For peer review only

ACKNOWLEDGEMENTS

The authors would like to acknowledge the Public Health England National Cancer Registration and Analysis Service (South West) for their assistance with this study.

FUNDING

The CAP trial was support by Cancer Research UK and the UK Department of Health (C11043/A4286, C18281/A8145, C18281/A11326 and C18281/A15064).

DATA SHARING STATEMENT

The dataset supporting the conclusions of this article cannot be shared with third parties due to conditions within the Section 251 approval that governs this research.

CONFLICTS OF INTEREST

All authors have completed the ICMJE uniform disclosure form at www.icmje.org/coi_disclosure.pdf and declare: no support from any organisation for the submitted work; RM,ET, JOD, and CM have received grants from Cancer Research UK in the previous three years; no other relationships or activities that could appear to have influenced the submitted work.

ETHICAL APPROVAL

Ethical approval to access information about all those in the CAP study was obtained from UK Trent Multi-Centre Research Ethics Committee (MREC/03/4/093). The CAP study was exempt from gaining individual consent to participant having obtained Section 251 approval from the UK Patient Information Advisory Group (PIAG) (now the Confidentiality Advisory Group, CAG), under Section 251 of the NHS Act 2006 [PIAG 4-09 (k)/2003]. The CAP study is sponsored by the University of Bristol and is registered at Current Controlled Trials (ISRCTN92187251).

AUTHORS CONTRIBUTIONS

The CAP trial was performed by R Martin, J Donovan, F Hamdy, R Neal, E Turner, C Metcalfe, G Young and E Walsh. E Turner and E Walsh extracted the relevant CAP trial data for this study. L Hounsome and I Tudge extracted the relevant NCRAS

For peer review only

registry data for this study. S Merriel, E Walsh and G Young performed the data analysis. S Merriel drafted this manuscript, to which all authors made amendments and approved the final version.

For peer review only

REFERENCES

1 Bray F, Parkin DM. Evaluation of data quality in the cancer registry: Principles and methods. Part I: Comparability, validity and timeliness. *European Journal of Cancer* 2009;**45**:747–55. doi:10.1016/j.ejca.2008.11.032

2 Parkin DM, Bray F. Evaluation of data quality in the cancer registry: Principles and methods Part II. Completeness. *European Journal of Cancer* 2009;**45**:756–64. doi:10.1016/j.ejca.2008.11.033

3 ONS. Cancer Registration Statistics, England: 2014. 2016;:1–17.

4 NCRAS. National Cancer Research and Analysis Service. httpwww.ncras.nhs.uk.

5 Department of Health. Cancer reform strategy. London: : Department of Health 2007.

6 Department of Health. Improving Outcomes: A Strategy for Cancer. 2011.

7 NCIN. Background to the Cancer Outcomes and Services Dataset (COSD). NCIN 2014.

8 West Midlands Cancer Intelligence Unit. Quantifying the Completeness of National Breast Cancer Data (cases diagnosed in 2006) Executive Summary. Breast Cancer Clinical Outcomes Measures 2009.

9 Turner EL, Metcalfe C, Donovan JL, *et al.* Design and preliminary recruitment results of the Cluster randomised triAl of PSA testing for Prostate cancer (CAP). *British Journal of Cancer* 2014;**110**:2829–36. doi:10.1038/bjc.2014.242

10 AJCC. Prostate Cancer Staging. American Cancer Society 2010.

11 Heidenreich A, Bastian PJ, Bellmunt J, *et al.* EAU Guidelines on Prostate Cancer. Part 1: Screening, Diagnosis, and Local Treatment with Curative Intent—Update 2013. *European Urology* 2014;**65**:124–37. doi:10.1016/j.eururo.2013.09.046

12 Epstein JI. Review Articles An Update of the Gleason Grading System. *JURO* 2010;**183**:433–40. doi:10.1016/j.juro.2009.10.046

13 UKIACR. Performance indicators. UKIACR. http://www.ukiacr.org/kpis (accessed 3 May2017).

14 Kearney TM, Donnelly C, Kelly JM, *et al.* Validation of the completeness and accuracy of the Northern Ireland Cancer Registry. *Cancer Epidemiology* 2015;**39**:401–4. doi:10.1016/j.canep.2015.02.005

15 Barlow L, Westergren K, Holmberg L, *et al.* The completeness of the Swedish Cancer Register – a sample survey for year 1998. *Acta*

For peer review only

Oncologica 2009;**48**:27–33. doi:10.1080/02841860802247664

16 Tomic K, Sandin F, Wigertz A, *et al.* Evaluation of data quality in the National Prostate Cancer Register of Sweden. *European Journal of Cancer* 2015;**51**:101–11. doi:10.1016/j.ejca.2014.10.025

17 Dimitrova N, Parkin DM. Data quality at the Bulgarian National Cancer Registry: An overview of comparability, completeness, validity and timeliness. *Cancer Epidemiology* 2015;**39**:405–13. doi:10.1016/j.canep.2015.03.015

18 Bakken IJ, Gystad SO, Christensen OOS, *et al.* Comparison of data from the Norwegian Patient Register and the Cancer Registry of Norway. *Tidsskrift for Den norske Legeforening* 2012;**132**:1336–40.

19 Kelloff GJ, Choyke P, Coffey DS, *et al.* Challenges in Clinical Prostate Cancer: Role of Imaging. *American Journal of Roentgenology* 2009;**192**:1455–70. doi:10.2214/AJR.09.2579

For peer review only

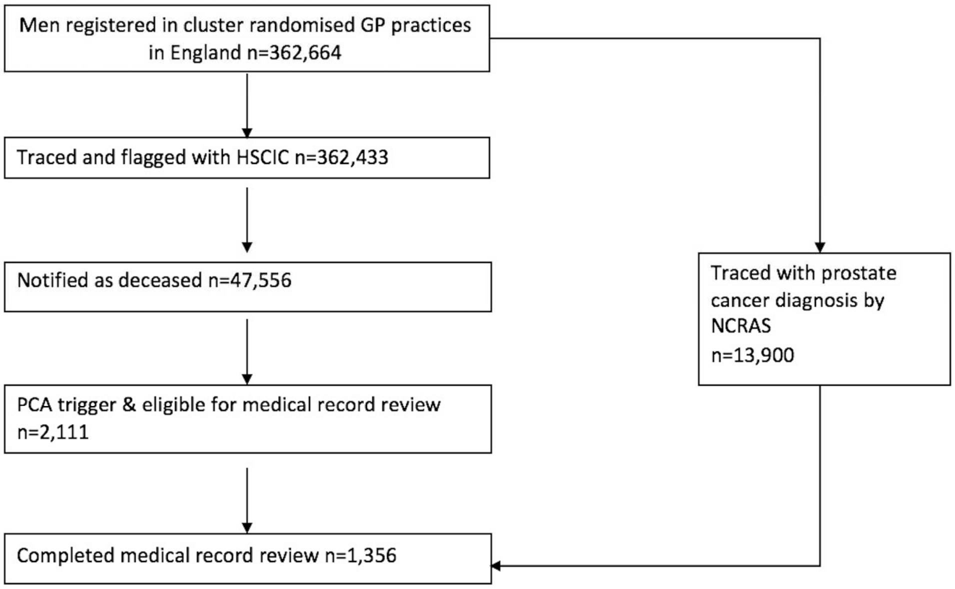

Figure 1 - Participant recruitment flow diagram

192x119mm (300 x 300 DPI)

STROBE Statement—Checklist of items that should be included in reports of ***cross-sectional studies***

	Item No	Recommendation
**Title and abstract**	1	*(a)* Indicate the study's design with a commonly used term in the title or the abstract<br>Page 1, Line 6
		(b) Provide in the abstract an informative and balanced summary of what was done and what was found<br>Page 2, Line 3
**Introduction**		
Background/rationale	2	Explain the scientific background and rationale for the investigation being reported<br>Page 5, Line 3-49
Objectives	3	State specific objectives, including any prespecified hypotheses<br>Page 5, Line 53-56
**Methods**		
Study design	4	Present key elements of study design early in the paper<br>Page 6, Line 37
Setting	5	Describe the setting, locations, and relevant dates, including periods of recruitment, exposure, follow-up, and data collection<br>Page 6, Line 6-37
Participants	6	*(a)* Give the eligibility criteria, and the sources and methods of selection of participants<br>Page 6, Line 6-37 & Figure 1
Variables	7	Clearly define all outcomes, exposures, predictors, potential confounders, and effect modifiers. Give diagnostic criteria, if applicable<br>Page 6, Line 59-58 & Page 7, Line 3-37
Data sources/ measurement	8*	For each variable of interest, give sources of data and details of methods of assessment (measurement). Describe comparability of assessment methods if there is more than one group<br>Page 6, Line 6-37
Bias	9	Describe any efforts to address potential sources of bias<br>Page 7, Line 29-37 & Page 8, Line 58 & Page 9, Line 3-4
Study size	10	Explain how the study size was arrived at<br>Page 6, Line 33-37
Quantitative variables	11	Explain how quantitative variables were handled in the analyses. If applicable, describe which groupings were chosen and why<br>Page 6, Line 58 & Page 3, Line 3-10 & Page 7, Line 29-37 Line 54-58 & Page 8, Line 3-4
Statistical methods	12	*(a)* Describe all statistical methods, including those used to control for confounding<br>Page 7, Line 47-58 & Page 8, Line 3-4
		(b) Describe any methods used to examine subgroups and interactions<br>Page 7, Line 54-58 & Page 8, Line 3-4
		(c) Explain how missing data were addressed<br>Page 7, Line 6-10, Line 21-26, Line 30-37
		(d) If applicable, describe analytical methods taking account of sampling strategy
		(*e*) Describe any sensitivity analyses
**Results**		
Participants	13*	(a) Report numbers of individuals at each stage of study—eg numbers potentially

For peer review only - http://bmjopen.bmj.com/site/about/guidelines.xhtml

		eligible, examined for eligibility, confirmed eligible, included in the study, completing follow-up, and analysed<br>See Figure 1
		(b) Give reasons for non-participation at each stage<br>See Figure 1
		(c) Consider use of a flow diagram<br>See Figure 1
Descriptive data	14*	(a) Give characteristics of study participants (eg demographic, clinical, social) and information on exposures and potential confounders<br>Page 9, Line 5-6
		(b) Indicate number of participants with missing data for each variable of interest<br>Page 9, Line 10-39 & Table 1
Outcome data	15*	Report numbers of outcome events or summary measures<br>Page 9, Line 10-39 & Table 1
Main results	16	*(a)* Give unadjusted estimates and, if applicable, confounder-adjusted estimates and their precision (eg, 95% confidence interval). Make clear which confounders were adjusted for and why they were included<br>Page 9, Line 28-58 & Page 10 Line 3-6 & Tables 2 and 3
		(b) Report category boundaries when continuous variables were categorized<br>Tables 2 and 3
		(*c*) If relevant, consider translating estimates of relative risk into absolute risk for a meaningful time period
Other analyses	17	Report other analyses done—eg analyses of subgroups and interactions, and sensitivity analyses
**Discussion**		
Key results	18	Summarise key results with reference to study objectives<br>Page 14, Line 5-17
Limitations	19	Discuss limitations of the study, taking into account sources of potential bias or imprecision. Discuss both direction and magnitude of any potential bias<br>Page 14, Line 45-53 & Page 15, Line 3-11
Interpretation	20	Give a cautious overall interpretation of results considering objectives, limitations, multiplicity of analyses, results from similar studies, and other relevant evidence<br>Page 15, Line 15-29
Generalisability	21	Discuss the generalisability (external validity) of the study results<br>Page 14 Line 20-42
**Other information**		
Funding	22	Give the source of funding and the role of the funders for the present study and, if applicable, for the original study on which the present article is based<br>Page 16, Line 12-13

*Give information separately for exposed and unexposed groups.

Note: An Explanation and Elaboration article discusses each checklist item and gives methodological background and published examples of transparent reporting. The STROBE checklist is best used in conjunction with this article (freely available on the Web sites of PLoS Medicine at http://www.plosmedicine.org/, Annals of Internal Medicine at http://www.annals.org/, and Epidemiology at http://www.epidem.com/). Information on the STROBE Initiative is available at www.strobe-statement.org.

For peer review only

BMJ Open

Cross-sectional study evaluating data quality of the National Cancer Registration and Analysis Service (NCRAS) prostate cancer registry data using the Cluster randomised triAl of PSA testing for Prostate cancer (CAP)

Journal:	*BMJ Open*
Manuscript ID	bmjopen-2017-015994.R2
Article Type:	Research
Date Submitted by the Author:	07-Jun-2017
Complete List of Authors:	Merriel, Samuel; University of Bristol School of Social and Community Medicine, Centre for Academic Primary Care<br>Turner, Emma; University of Bristol, School of Social and Community Medicine<br>Walsh, Eleanor; University of Bristol, School of Social and Community Medicine<br>Young, Grace; University of Bristol, School of Social and Community Medicine<br>Metcalfe, Chris; University of Bristol, Social Medicine<br>Hounsome, Luke; Public Health England South Region, National Cancer Registration and Analysis Service<br>Tudge, Isobel; Public Health England South Region, National Cancer Registry and Analysis Service<br>Donovan, Jenny; University of Bristol<br>Hamdy, Freddie; University of Oxford, Nuffield Department of Surgery<br>Neal, David; University of Oxford Nuffield Department of Surgical Sciences<br>Martin, Richard; University of Bristol, Social Medicine
**Primary Subject Heading**:	Epidemiology
Secondary Subject Heading:	Oncology, Urology, Public health
Keywords:	Prostate disease < UROLOGY, Epidemiology < ONCOLOGY, Urological tumours < ONCOLOGY

SCHOLARONE™
Manuscripts

Cross-sectional study evaluating data quality of the National Cancer Registration and Analysis Service (NCRAS) prostate cancer registry data using the Cluster randomised triAl of PSA testing for Prostate cancer (CAP)

Dr Samuel WD Merriel[1], Dr Emma L Turner[1], Ms Eleanor Walsh[1], Ms Grace J Young[1], Dr Chris Metcalfe[1], Dr Luke Hounsome[2], Ms Isobel Tudge[2], Prof Jenny Donovan[1], Prof Freddie Hamdy[3], Prof David Neal[3], Prof Richard M Martin[1]

[1]School of Social & Community Medicine, University of Bristol, Bristol, UK

[2]Public Health England National Cancer Registration and Analysis Service, Bristol, UK

[3]Nuffield Department of Surgical Sciences, Oxford University, Oxford, UK

Corresponding author

Dr Samuel William David Merriel

Centre for Academic Primary Care

School of Social & Community Medicine

University of Bristol

39 Whatley Road

Clifton, Bristol, UK

Sam.merriel@bristol.ac.uk

0117 928 9000

Keywords – prostate cancer, registry, validation

Word count – 2065

For peer review only

ABSTRACT (296/300)

Objectives

To compare the completeness and agreement of prostate cancer data recorded by the National Cancer Registration and Analysis Service (NCRAS) with research-level data specifically abstracted from medical records from the Cluster randomised trial of PSA testing for Prostate cancer (CAP) trial.

Design

Cross-sectional comparison study

Participants

We included 1,356 men from the CAP trial cohort who were linked to the NCRAS registry.

Primary and secondary outcome measures

Completeness of prostate cancer data in NCRAS and CAP and agreement for TNM stage (T1/T2; T3; T4/N1/M1) and Gleason grade (4-6; 7; 8-10), measured by differences in proportions and Cohen's Kappa statistic. Data were also stratified by year and pre- versus post-2010, when NCRAS reporting standards changed.

Results

Compared to CAP, completeness was lower in NCRAS for Gleason grade (41.2% vs 76.7%, difference 35.5 95% CI 32.1, 39.0) and TNM stage (29.9% vs 67.6%, difference 37.6 95% CI 34.1, 41.1). NCRAS completeness for Gleason grade (pre- versus post-2010 31.69% vs 64%; difference 32.31 95% CI 26.76, 37.87) and TNM stage (19.31% vs 55.50%; difference 36.19 95% CI 30.72, 41.67) improved over time. Agreement for Gleason grade was high (Cohen's Kappa, κ=0.90 95% CI 0.88, 0.93), but lower for TNM stage (κ=0.41 95% CI 0.37, 0.51) overall. There was a trend towards improved agreement on Gleason grade, but not TNM stage, when comparing pre- and post-2010.

Conclusion

NCRAS case identification was very high, however data on prostate cancer grade was less complete than CAP, and agreement for TNM stage was modest. Although the completeness of NCRAS data has improved since 2010, the higher completeness rate in CAP demonstrate that gains could potentially be achieved in routine registry data.

For peer review only

This study's findings highlight a need for improved recording of stage and grade data in the source medical records.

For peer review only

ARTICLE SUMMARY

Strengths and limitations of this study

- Cross-sectional study design
- Comparison of English prostate cancer registry with independently collected, research-level data from a randomised controlled trial on prostate cancer screening
- Low level of completeness of TNM staging data in NCRAS overall
- Reporting standards for NCRAS registry changed in 2010, affecting the consistency of data collection and quality across the study period

For peer review only

INTRODUCTION

Accurate and complete cancer registry data can inform health policy, prioritise the allocation of limited funds available for cancer prevention and treatment, and be used for research purposes. Bray and Parkin[1,2] outline methods for evaluating the quality of cancer registry data, including comparisons with other local cancer registries, routinely collected data, large-scale research cohorts, or estimates using incidence rates.

Prostate cancer is the most common cancer in males in England, with 39,741 new cases registered in 2014[3]. The National Cancer Registration and Analysis Service (NCRAS) collects data on new cases of cancer in England[4], including prostate cancer. The NCRAS was established in February 2016 to incorporate the National Cancer Registry Service and the National Cancer Intelligence Network, and is now part of Public Health England. The 'Cancer reform strategy'[5] and the 'Improving outcomes: a strategy for cancer'[6] reports from the Department of Health in England highlighted the need for better information and higher quality data to support efforts to improve cancer outcomes. The National Cancer Dataset was subsequently replaced by the Cancer Outcomes and Services Dataset to improve cancer registry data collection standards in England [7]. There is some gray literature on the quality of the NCRAS cancer registry data, showing low levels of completeness of TNM stage[8]. However, to our knowledge, comparison studies against independently collected data have not yet been published for prostate cancer.

The **C**luster randomised tri**A**l of PSA testing for **P**rostate cancer (CAP) trial is a population-based cluster randomized controlled trial (RCT) in England and Wales assessing the impact of a single invitation for PSA testing for prostate cancer on prostate cancer mortality[9]. Within this trial, trained researchers extract detailed data from the medical records of men who have died with or of prostate cancer onto a structured proforma. These data offered the opportunity to compare the completeness and agreement of the NCRAS cancer registry data with an independent research-level data-source.

For peer review only

MATERIALS AND METHODS

Participant selection

NCRAS collate patient-identifiable demographic and cancer specific clinical information based on the Clinical Outcomes and Services Dataset from NHS service providers, unless the patient opts-out.

The CAP trial involves 416,387 men aged 50-69 years from 271 primary care (GP) practices in England and Wales that were randomised between 2001 and 2007[9]. In this trial these men were flagged with the Health & Social Care Information Centre (HSCIC) to trace cancer and death registrations. The comparison with the NCRAS data restricts the analysis to those from CAP practices in England who were deceased. In England, 362,433 CAP men were successfully traced by the HSCIC and 47,556 were notified as having died of any cause. Of the men successfully traced and who had died, 2,111 were identified from HSCIC data as having a prostate cancer diagnosis or having prostate cancer listed anywhere on the death certificate. These men underwent medical record review by specially trained CAP research staff to ascertain cause of death independent from information held on the death certificate and the cancer registry[9]. At the time of this analysis, 1,356 medical record reviews had been completed for men diagnosed with prostate cancer between February 2002 and December 2015, who had died between April 2003 and October 2015. These men were matched to their NCRAS dataset entry using their NHS number (see figure 1), then given a unique identifier and merged.

Figure 1 – Participant recruitment flow diagram

Data

The date of birth provided by the GP practices at the time of recruitment was used to calculate age at entry into the CAP study and age at diagnosis.

The NCRAS team extracted available diagnostic data including date of diagnosis, American Joint Committee on Cancer[10] and Union Internationale Contre le Cancer (UICC) TNM staging[11] and Gleason grade[12] for the current analysis. Clinical TNM

For peer review only

stage was derived from available imaging data, and pathological TNM stage was derived from pathology reports of tissue samples if surgery was within 6 months of diagnosis, with final TNM staging altered if necessary. NCRAS Clinical TNM stage data was combined with pathological stage data by the NCRAS team where it was incomplete to produce a 'best' stage, and these data were used in the analysis.

The data collected for CAP from the medical records included: date of prostate cancer diagnosis, diagnostic Gleason grade and diagnostic clinical UICC TNM stage. These data were extracted for the current analysis, as well as digital rectal examination (DRE) findings, radiology reports and PSA blood results at the time of diagnosis. The results of these investigations were used in the current analysis to derive clinical TNM stage if this was missing from the data extraction. Diagnostic clinical stage, grade and other investigations were selected by taking the closest record to the date of diagnosis within +/-6 months.

TNM stage data gathered for CAP did not always yield a complete TxNxMx stage from the clinical record. A pragmatic, cascading hierarchical approach was therefore utilised if this situation occurred. Any evidence of advanced disease (T4 or N1 or M1) was gathered initially, followed by evidence of locally advanced disease (T3), then localised disease (T1 or T2). This was done separately for NCRAS and CAP stage data.

This study follows the STROBE reporting guidelines for observational studies (STROBE checklist submitted as Supplementary file).

Statistical analysis

All analyses were performed using STATA 14 (StataCorp, 2015). We compared the completeness of Gleason grade and TNM stage between NCRAS and CAP data using differences in proportions (with 95% confidence intervals). The agreement between the NCRAS data and CAP data was assessed using Cohen's Kappa statistic, with 95% confidence intervals, for Gleason grading and TNM staging. We also undertook an analysis comparing localised (T1/2) vs locally advanced (T3) vs advanced (T4 or N1 or M1) prostate cancer. Reporting standards for new cases to the registry changed in

For peer review only

2010-2011[7], so the completeness data was also stratified by year of prostate cancer diagnosis and 2002-2009 vs 2010-2015.

For peer review only

RESULTS

The average age at the time of diagnosis of both the NCRAS and CAP participants was 75.2 years (SD 5.1).

97.9% (n=1,327) of men with prostate cancer notified to the NCRAS registry had a complete diagnosis date. Gleason grade was entered for 41.2% (95% CI 38.6, 43.8) of NCRAS men. UICC TNM stage was entered for 29.9% (95% CI 27.5, 32.5). (**See Table 1**).

Of the CAP trial men with complete medical record reviews, 98.5% (n=1,336) had complete date of birth and diagnosis date. The overall completeness of Gleason grade data and TNM stage was 76.7% (95% CI 74.4, 78.9) and 67.6% (95% CI 65.0, 70.0) respectively.

The date of diagnosis in the NCRAS registry exactly matched CAP trial men in 44.5% (n=583) of cases. The median difference in diagnosis date for NCRAS patients was 0 days (interquartile range: 0, 7) later than CAP men. NCRAS had significantly lower overall completeness than CAP for Gleason grade (difference in proportions 35.6% 95% CI 32.1, 39.0), and TNM stage (37.6% 95% CI 34.1, 41.1), although the difference in completeness of Gleason grade (1.00% 95% CI -5.63, 7.63) and TNM stage (11.75% 95% CI 5.05, 18.45) data was much smaller from 2010 onwards.

Table 2 shows agreement on Gleason grade data in the registry and the CAP trial, which was strong. Agreement was high for combined (κ=0.90 95% CI 0.89, 0.92) Gleason grade overall. There was a trend towards higher agreement in 2010-2015 compared to 2002-2009 for low, moderate and high combined Gleason grade. This level of agreement was maintained across individual levels of primary (κ=0.84 95% CI 0.82, 0.86) and secondary Gleason grades (κ=0.80 95% CI 0.79, 0.83).

Table 3 shows agreement on overall UICC TNM staging. The levels of agreement for overall stage accuracy were weak to moderate. Similar levels of agreement were found when considering the data categorized as localised (T1/2 k=0.53 95% CI 0.42,

For peer review only

0.64), locally advanced (T3 k= 0.29 95% CI 0.17, 0.41) and advanced (T4 or N1 or M1 k=0.47 95% CI 0.37, 0.57). These findings were consistent when comparing data from 2002 – 2009 with 2010 – 2015.

For peer review only

n = 1356	Combined Gleason grade						TNM stage					
	**NCRAS**		**CAP**		**Diff**	**95%CI**	**NCRAS**		**CAP**		**Diff**	**95%CI**
2002-2003	22	26.19%	74	88.10%	-61.91%	-73.58, -50.23	7	8.33%	56	66.67%	-58.33%	-70.02, -46.65
2004	25	33.33%	59	78.67%	-45.34%	-59.47, -31.20	18	24.00%	50	66.67%	-42.67%	-57.06, -28.27
2005	41	33.61%	101	82.79%	-49.18%	-59.91, -38.45	30	24.59%	84	68.89%	-44.26%	-55.48, -33.04
2006	47	29.94%	129	82.17%	-52.23%	-61.57, -42.89	21	13.38%	109	69.43%	-56.05%	-65.01, -47.09
2007	55	31.43%	150	85.71%	-54.28%	-62.90, -45.67	33	18.86%	128	73.14%	-54.29%	-63.04, -45.53
2008	63	34.05%	157	84.86%	-50.81%	-59.37, -42.25	32	17.30%	114	61.62%	-44.32%	-53.20, -35.45
2009	49	31.61%	110	70.97%	-39.36%	-49.58, -29.13	43	27.74%	106	68.39%	-40.65%	-50.81, -30.48
2010	93	62.84%	105	70.95%	-8.11%	-18.79, 2.57	53	35.81%	89	60.16%	-24.32%	-35.36, -13.28
2011	77	68.14%	73	64.60%	3.54%	-8.77, 15.85	51	45.13%	84	74.34%	-29.20%	-41.41, -16.99
2012	66	64.71%	59	57.84%	6.87%	-6.47, 20.20	90	88.24%	69	67.65%	20.59%	9.56, 31.61
2013-2015	20	54.05%	23	62.16%	-8.11%	-30.51, 14.30	28	75.68%	27	72.97%	2.70%	-17.19, 22.60
2002-2009	302	31.69%	780	81.85%	-50.16%	-53.99, -46.32	184	19.31%	647	67.89%	-48.48%	-52.47, -44.70
2010 - 2015	256	64.00%	260	65.00%	-1.00%	-7.63, 5.63	222	55.50%	269	67.25%	-11.75%	-18.45, -5.05
**Overall**	558	41.15%	1040	76.70%	-35.55%	-38.99, -32.09	406	29.94%	916	67.55%	-37.61%	-41.10, -34.12

Table 1 - Completeness of Gleason grade and TNM stage in NCRAS registry and CAP (n, %)

For peer review only - http://bmjopen.bmj.com/site/about/guidelines.xhtml

n=1356	**NCRAS (n)**	**CAP (n)**	**Kappa (k)**	**95% CI**
Combined Gleason grade (2002 – 2009)				
4-6	80	202	0.84	0.77, 0.92
7	97	241	0.86	0.79, 0.92
8-10	125	337	0.92	0.88, 0.97
Overall	302	780	0.87	0.84, 0.92
Combined Gleason grade (2010 – 2015)				
4-6	37	28	0.95	0.89, 1.00
7	90	93	0.95	0.90, 0.99
8-10	129	139	0.97	0.93, 0.99
Overall	256	260	0.93	0.92, 0.94
Combined Gleason grade (overall)				
4-6	117	230	0.88	0.83, 0.94
7	187	334	0.90	0.86, 0.94
8-10	254	476	0.94	0.92, 0.97
Overall	558	1040	0.90	0.88, 0.93

Table 2 - Agreement between NCRAS registry and CAP on combined Gleason grade

For peer review only

n = 1356	**NCRAS (n)**	**CAP (n)**	**Kappa (k)**	**95% CI**
TNM stage (2002 – 2009)				
T1/T2	91	218	0.55	0.40, 0.69
T3	21	242	0.33	0.15, 0.51
T4/N1/M1	72	122	0.53	0.38, 0.67
Overall	184	582	0.47	0.43, 0.50
TNM stage (2010 – 2015)				
T1/T2	64	57	0.45	0.28, 0.62
T3	40	90	0.25	0.08, 0.41
T4/N1/M1	117	84	0.41	0.28, 0.55
Overall	221	231	0.34	0.27, 0.37
TNM stage (overall)				
T1/T2	155	275	0.53	0.42, 0.64
T3	61	332	0.29	0.17, 0.41
T4/N1/M1	189	206	0.47	0.37, 0.57
Overall	405	813	0.41	0.37, 0.51

Table 3 - Agreement between NCRAS registry and CAP on TNM stage

For peer review only

DISCUSSION

There were mixed levels of completeness and agreement of stage and grade information comparing NCRAS with CAP research-level data. We showed a high level of completeness for case identification and diagnosis dates in both cohorts. NCRAS completeness for Gleason grade was moderate and TNM stage completeness was low. From 2010 the completeness of stage and grade data in the NCRAS registry increased. Completeness in CAP remained consistently high throughout the study period. Agreement between NCRAS and CAP was high for Gleason grade and moderate for TNM stage.

The completeness of case capture and diagnosis date are consistent with the UK and Ireland Association of Cancer Registries (UKIACR) most recent performance indicators.[13] Our results are similar to those from validation studies of cancer registries in other countries. Estimates of completeness for registries in Ireland[14], Sweden[15,16], Bulgaria[17], and Norway[18] were generally high, ranging from 91% - 97%. These studies compared registry data with other population registries or primary care records. None of them used an independently collected research-level cancer trial data set for comparison. Tomic et al[16] found a higher level of agreement in Sweden for both TNM staging (83%) and Gleason grading (97%) when compared to our study. This could be due to the lower level of grade and stage completeness in our study and changes in the UICC TNM classification during the study period, and the fact that Tomic et al compared two cancer registries that gathered prostate cancer data on the same population.

The strengths of the data used in the current analysis to compare completeness and agreement with the NCRAS cancer registry were that they were obtained through extensive medical notes review by trained researchers. Further, almost every CAP trial man's diagnosis had been notified to the NCRAS registry, allowing a thorough comparative analysis.

The completeness of UICC TNM stage data in the registry was moderate, making it more difficult to draw conclusions about the accuracy of TNM staging. A possible

For peer review only

reason for differences in agreement between NCRAS and CAP could relate to the fact that NCRAS more commonly partially or completely used pathological TNM staging compared to CAP. Distinguishing TNM stage of prostate cancer radiologically, particularly T1b vs T2, is a an evolving area[19], and this may explain some of the difficulties in reporting. Reporting standards for NCRAS cancer registries changed in 2010 and completeness improved after the changes were made[7].

Our findings suggest that prostate cancer data in the NCRAS cancer registry is complete in terms of identifying and recording new cases. Whilst the agreement for Gleason grade was high, the completeness and agreement of TNM stage data was lower for the years covered in this analysis, highlighting a need for improved recording of these data in the source medical records. This study also demonstrates how trial data can be verified for completeness and accuracy using empirical data. Completeness of NCRAS data has improved since 2010, however the higher completeness rate in CAP highlights what further gains could potentially be achieved in routine registry data. Complete and accurate national cancer registries are vital in order to inform health policy, healthcare spending, and research.

For peer review only

ACKNOWLEDGEMENTS

The authors would like to acknowledge the Public Health England National Cancer Registration and Analysis Service (South West) for their assistance with this study.

FUNDING

The CAP trial was support by Cancer Research UK and the UK Department of Health (C11043/A4286, C18281/A8145, C18281/A11326 and C18281/A15064).

DATA SHARING STATEMENT

The dataset supporting the conclusions of this article cannot be shared with third parties due to conditions within the Section 251 approval that governs this research.

CONFLICTS OF INTEREST

All authors have completed the ICMJE uniform disclosure form at www.icmje.org/coi_disclosure.pdf and declare: no support from any organisation for the submitted work; RM,ET, JOD, and CM have received grants from Cancer Research UK in the previous three years; no other relationships or activities that could appear to have influenced the submitted work.

ETHICAL APPROVAL

Ethical approval to access information about all those in the CAP study was obtained from UK Trent Multi-Centre Research Ethics Committee (MREC/03/4/093). The CAP study was exempt from gaining individual consent to participant having obtained Section 251 approval from the UK Patient Information Advisory Group (PIAG) (now the Confidentiality Advisory Group, CAG), under Section 251 of the NHS Act 2006 [PIAG 4-09 (k)/2003]. The CAP study is sponsored by the University of Bristol and is registered at Current Controlled Trials (ISRCTN92187251).

AUTHORS CONTRIBUTIONS

The CAP trial was performed by R Martin, J Donovan, F Hamdy, R Neal, E Turner, C Metcalfe, G Young and E Walsh. E Turner and E Walsh extracted the relevant CAP trial data for this study. L Hounsome and I Tudge extracted the relevant NCRAS

For peer review only

registry data for this study. S Merriel, E Walsh and G Young performed the data analysis. S Merriel drafted this manuscript, to which all authors made amendments and approved the final version.

For peer review only

REFERENCES

1 Bray F, Parkin DM. Evaluation of data quality in the cancer registry: Principles and methods. Part I: Comparability, validity and timeliness. *European Journal of Cancer* 2009;**45**:747–55. doi:10.1016/j.ejca.2008.11.032

2 Parkin DM, Bray F. Evaluation of data quality in the cancer registry: Principles and methods Part II. Completeness. *European Journal of Cancer* 2009;**45**:756–64. doi:10.1016/j.ejca.2008.11.033

3 ONS. Cancer Registration Statistics, England: 2014. 2016;:1–17.

4 NCRAS. National Cancer Research and Analysis Service. httpwww.ncras.nhs.uk.

5 Department of Health. Cancer reform strategy. London: : Department of Health 2007.

6 Department of Health. Improving Outcomes: A Strategy for Cancer. 2011.

7 NCIN. Background to the Cancer Outcomes and Services Dataset (COSD). NCIN 2014.

8 West Midlands Cancer Intelligence Unit. Quantifying the Completeness of National Breast Cancer Data (cases diagnosed in 2006) Executive Summary. Breast Cancer Clinical Outcomes Measures 2009.

9 Turner EL, Metcalfe C, Donovan JL, *et al.* Design and preliminary recruitment results of the Cluster randomised triAl of PSA testing for Prostate cancer (CAP). *British Journal of Cancer* 2014;**110**:2829–36. doi:10.1038/bjc.2014.242

10 AJCC. Prostate Cancer Staging. American Cancer Society 2010.

11 Heidenreich A, Bastian PJ, Bellmunt J, *et al.* EAU Guidelines on Prostate Cancer. Part 1: Screening, Diagnosis, and Local Treatment with Curative Intent—Update 2013. *European Urology* 2014;**65**:124–37. doi:10.1016/j.eururo.2013.09.046

12 Epstein JI. Review Articles An Update of the Gleason Grading System. *JURO* 2010;**183**:433–40. doi:10.1016/j.juro.2009.10.046

13 UKIACR. Performance indicators. UKIACR. http://www.ukiacr.org/kpis (accessed 3 May2017).

14 Kearney TM, Donnelly C, Kelly JM, *et al.* Validation of the completeness and accuracy of the Northern Ireland Cancer Registry. *Cancer Epidemiology* 2015;**39**:401–4. doi:10.1016/j.canep.2015.02.005

15 Barlow L, Westergren K, Holmberg L, *et al.* The completeness of the Swedish Cancer Register – a sample survey for year 1998. *Acta*

For peer review only

Oncologica 2009;**48**:27–33. doi:10.1080/02841860802247664

16 Tomic K, Sandin F, Wigertz A, *et al.* Evaluation of data quality in the National Prostate Cancer Register of Sweden. *European Journal of Cancer* 2015;**51**:101–11. doi:10.1016/j.ejca.2014.10.025

17 Dimitrova N, Parkin DM. Data quality at the Bulgarian National Cancer Registry: An overview of comparability, completeness, validity and timeliness. *Cancer Epidemiology* 2015;**39**:405–13. doi:10.1016/j.canep.2015.03.015

18 Bakken IJ, Gystad SO, Christensen OOS, *et al.* Comparison of data from the Norwegian Patient Register and the Cancer Registry of Norway. *Tidsskrift for Den norske Legeforening* 2012;**132**:1336–40.

19 Kelloff GJ, Choyke P, Coffey DS, *et al.* Challenges in Clinical Prostate Cancer: Role of Imaging. *American Journal of Roentgenology* 2009;**192**:1455–70. doi:10.2214/AJR.09.2579

For peer review only

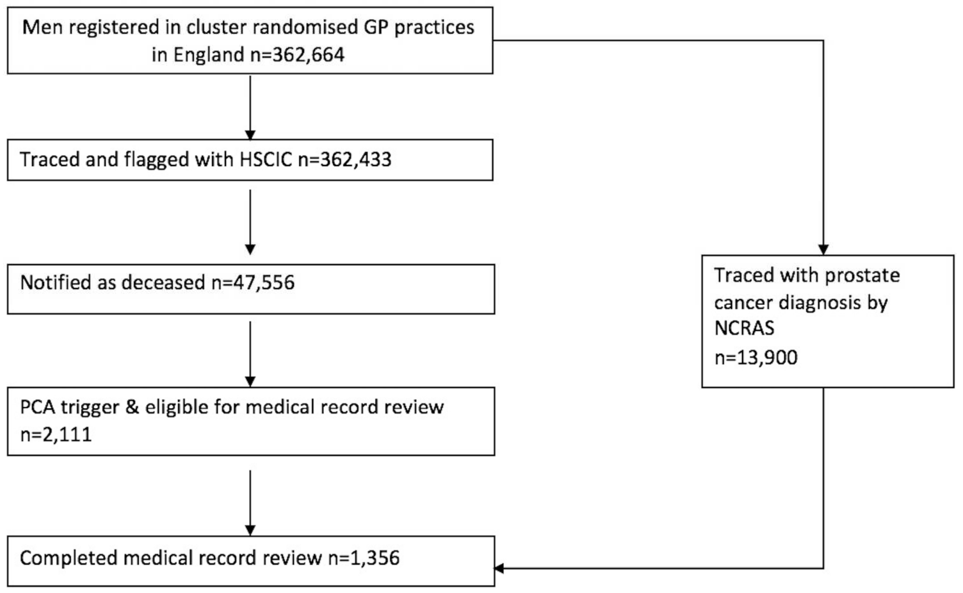

Figure 1 - Participant recruitment flow diagram

192x119mm (300 x 300 DPI)

STROBE Statement—Checklist of items that should be included in reports of ***cross-sectional studies***

	Item No	Recommendation
**Title and abstract**	1	*(a)* Indicate the study's design with a commonly used term in the title or the abstract<br>Page 1, Line 6
		(b) Provide in the abstract an informative and balanced summary of what was done and what was found<br>Page 2, Line 3
**Introduction**		
Background/rationale	2	Explain the scientific background and rationale for the investigation being reported<br>Page 5, Line 3-49
Objectives	3	State specific objectives, including any prespecified hypotheses<br>Page 5, Line 53-56
**Methods**		
Study design	4	Present key elements of study design early in the paper<br>Page 6, Line 37
Setting	5	Describe the setting, locations, and relevant dates, including periods of recruitment, exposure, follow-up, and data collection<br>Page 6, Line 6-37
Participants	6	*(a)* Give the eligibility criteria, and the sources and methods of selection of participants<br>Page 6, Line 6-37 & Figure 1
Variables	7	Clearly define all outcomes, exposures, predictors, potential confounders, and effect modifiers. Give diagnostic criteria, if applicable<br>Page 6, Line 59-58 & Page 7, Line 3-37
Data sources/ measurement	8*	For each variable of interest, give sources of data and details of methods of assessment (measurement). Describe comparability of assessment methods if there is more than one group<br>Page 6, Line 6-37
Bias	9	Describe any efforts to address potential sources of bias<br>Page 7, Line 29-37 & Page 8, Line 58 & Page 9, Line 3-4
Study size	10	Explain how the study size was arrived at<br>Page 6, Line 33-37
Quantitative variables	11	Explain how quantitative variables were handled in the analyses. If applicable, describe which groupings were chosen and why<br>Page 6, Line 58 & Page 3, Line 3-10 & Page 7, Line 29-37 Line 54-58 & Page 8, Line 3-4
Statistical methods	12	*(a)* Describe all statistical methods, including those used to control for confounding<br>Page 7, Line 47-58 & Page 8, Line 3-4
		(b) Describe any methods used to examine subgroups and interactions<br>Page 7, Line 54-58 & Page 8, Line 3-4
		(c) Explain how missing data were addressed<br>Page 7, Line 6-10, Line 21-26, Line 30-37
		(d) If applicable, describe analytical methods taking account of sampling strategy
		(*e*) Describe any sensitivity analyses
**Results**		
Participants	13*	(a) Report numbers of individuals at each stage of study—eg numbers potentially

For peer review only - http://bmjopen.bmj.com/site/about/guidelines.xhtml

For peer review only

		eligible, examined for eligibility, confirmed eligible, included in the study, completing follow-up, and analysed<br>See Figure 1
		(b) Give reasons for non-participation at each stage<br>See Figure 1
		(c) Consider use of a flow diagram<br>See Figure 1
Descriptive data	14*	(a) Give characteristics of study participants (eg demographic, clinical, social) and information on exposures and potential confounders<br>Page 9, Line 5-6
		(b) Indicate number of participants with missing data for each variable of interest<br>Page 9, Line 10-39 & Table 1
Outcome data	15*	Report numbers of outcome events or summary measures<br>Page 9, Line 10-39 & Table 1
Main results	16	*(a)* Give unadjusted estimates and, if applicable, confounder-adjusted estimates and their precision (eg, 95% confidence interval). Make clear which confounders were adjusted for and why they were included<br>Page 9, Line 28-58 & Page 10 Line 3-6 & Tables 2 and 3
		(b) Report category boundaries when continuous variables were categorized<br>Tables 2 and 3
		(*c*) If relevant, consider translating estimates of relative risk into absolute risk for a meaningful time period
Other analyses	17	Report other analyses done—eg analyses of subgroups and interactions, and sensitivity analyses
**Discussion**		
Key results	18	Summarise key results with reference to study objectives<br>Page 14, Line 5-17
Limitations	19	Discuss limitations of the study, taking into account sources of potential bias or imprecision. Discuss both direction and magnitude of any potential bias<br>Page 14, Line 45-53 & Page 15, Line 3-11
Interpretation	20	Give a cautious overall interpretation of results considering objectives, limitations, multiplicity of analyses, results from similar studies, and other relevant evidence<br>Page 15, Line 15-29
Generalisability	21	Discuss the generalisability (external validity) of the study results<br>Page 14 Line 20-42
**Other information**		
Funding	22	Give the source of funding and the role of the funders for the present study and, if applicable, for the original study on which the present article is based<br>Page 16, Line 12-13

*Give information separately for exposed and unexposed groups.

Note: An Explanation and Elaboration article discusses each checklist item and gives methodological background and published examples of transparent reporting. The STROBE checklist is best used in conjunction with this article (freely available on the Web sites of PLoS Medicine at http://www.plosmedicine.org/, Annals of Internal Medicine at http://www.annals.org/, and Epidemiology at http://www.epidem.com/). Information on the STROBE Initiative is available at www.strobe-statement.org.

For peer review only
